# Patterns of Tau deposition in Alzheimer’s Disease

**DOI:** 10.1002/alz.095783

**Published:** 2025-01-09

**Authors:** Dhivya Srinivasan, Ilya M. Nasrallah, Zhijian Yang, Guray Erus, Christos Davatzikos

**Affiliations:** ^1^ Artificial Intelligence in Biomedical Imaging Laboratory (AIBIL), Center for and Data Science for Integrated Diagnostics (AI2D), Perelman School of Medicine, University of Pennsylvania, Philadelphia, PA USA; ^2^ Center for Biomedical Image Computing and Analytics, University of Pennsylvania, Philadelphia, PA USA; ^3^ University of Pennsylvania, Philadelphia, PA USA

## Abstract

**Background:**

Spread of tau tangles, one of the hallmarks of Alzheimer’s Disease (AD) is complex and heterogenous. While many studies focus on stereotypical patterns of tau, there are many individuals who deviates from Braak staging (ex.atypical tau). Understanding heterogeneity of tau spread and association with other factors has important implications in clinical trials. In this analysis, we derived four distinct patterns of tau using a data driven, deep learning‐based clustering method, Surreal‐GAN.

**Method:**

We used data from 1445 (Amyloid+:792, Amyloid‐:653) subjects with ^18^F‐flortaucipir PET from the Alzheimer’s Disease Neuroimaging Initiative (ADNI; N = 803), HABS; N = 195 and A4; N = 447. We used study‐defined cut‐offs for deriving amyloid status for each study separately. In this analysis, we utilized tau regional standardized uptake value ratios(SUVR) calculated for each of the aparc+aseg FreeSurfer labels. We then applied Surreal‐GAN on the regional tau SUVR for Amyloid + subjects with Amyloid ‐ as reference group to determine the Tau spatiotemporal subtypes. We tested the associations between r‐indices and cognitive scores.

**Result:**

Participants had a mean age of 71.8 (±4.8); 73.5(±6.2); 73.8(±7.6) years for A4, HABS and ADNI respectively. Surreal‐GAN showed optimal agreement indices for four patterns. Pattern r1 (r.occipital) shows the involvement of cuneus, lingual gyrus; pattern r2 (r.frontotemporal) shows frontal and temporal; pattern r3 (r.parietal) shows precuneus and inferior parietal and pattern r4 (r.typical) shows typical AD regions. Pattern r.occiptal was associated with language area of cognitive function(MMSE language domain); We observed significant associations of r.typical with composite memory scores in ADNI.

**Conclusion:**

Employing a data‐driven deep learning‐based approach, we derived more than one dissociable pattern of tau deposition. These findings could inform identification of subgroups of individuals who may or may not be responsive to particular clinical intervention.

